# On the crystal structure of tri­benzyl­tin(IV) iodide, Bz_3_SnI: a correction

**DOI:** 10.1107/S2414314626002142

**Published:** 2026-03-03

**Authors:** Nadia-Ammane Reuter, Hans Reuter

**Affiliations:** a Chemistry, Osnabrück University, Barabarstr. 7, 49069 Osnabrück, Germany

**Keywords:** crystal structure, bond lengths, dipole-dipole inter­actions, polar space group

## Abstract

Tri­benzyl­iodido­tin(IV), [Sn(C_7_H_7_)_3_I], has been prepared and its crystal structure determined in order to clarify some discrepancies in a previous report. The compound does not crystallize in the rhombohedral space group *R*3 as originally assumed [Wang *et al.* (2011 ▸). *Wuji Huaxue Xuebao*, **27**, 487–490], but in the monoclinic space group *Cc*, and also does not have a tin–iodide distance of 2.452 (3) but of 2.7165 (2) Å. Furthermore, the mol­ecules are not associated in strands *via* iodide bridges but are isolated from each other, with their dipole moments forming an angle of 23.53 (1)° to the *c*-glide plane of the space group.

## Abstract 2

Single crystals of tri­benzyl­tin(IV) iodide, prepared from hexa­benzyl­distannoxane, Bz_3_SnOSnBz_3_, and hydro­iodic acid, have been characterized by single-crystal X-ray diffraction in order to clarify some inconsistencies in the original structure determination. In particular, the present study provides a significantly longer tin–iodide distance, which corresponds much better with those in related structural studies.

## Graphical abstract

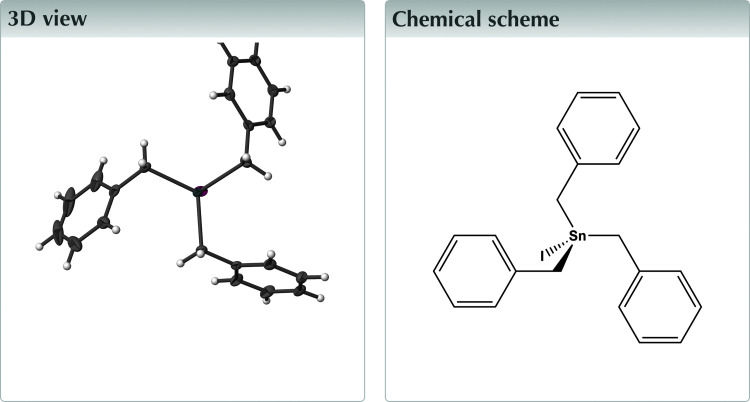

## Structure description

According to the Cambridge Structural Database (Groom *et al.*, 2016 ▸), the crystal structure of tri­benzyl­tin(IV) iodide, [Sn(C_7_H_7_)_3_I], was published by Wang *et al.* (2011 ▸). The associated deposition number is 796810 and the database identifier is ONIVAY. An English translation of the title and abstract of the article, which was written in Chinese, can be found *via SciFinder-n* (Chemical Abstract Service, 2026 ▸) among the references for the CA-number 19127–38-9. This shows that the compound is ‘tri­benzyl­tin(IV) iodide’, synthesized *via* the reaction of tri­benzyl­tin(IV) chloride with iodo­acetic acid. The reported data indicates that the compound crystallizes in the rhombohedral space group *R*3 with one mol­ecule in the unit cell.

At first glance, this appears to be a completely normal structure refinement, apart from the fact that the listed *R* value (0.072) is unusually high. After downloading the CIF file and analysing the data with a graphics program, it quickly becomes clear that something about the structure cannot be right. Although the mol­ecule has the umbrella-like structure already known from the corresponding chloride (Ng, 2009 ▸), the reported tin–iodide distance is too short [2.452 (3) Å]. In the literature, tin–iodide distances of 2.6916 (8)/2.7060 (8) Å (Simard & Warf, 1994 ▸), 2.7081 (6) Å (Ng, 1995 ▸) and 2.6758 (8) Å (Mao *et al.*, 2006 ▸) are found at ambient temperature in the three different modifications of Ph_3_SnI, while a value of 2.7463 (6) Å is observed in the case of tri­cyclo­hexyl­tin(IV) iodide at T = 120 K (Howie *et al.*, 2004 ▸). These values correlate quite well with the sum (2.76 Å) of the covalent radii (Cordero *et al.*, 2008 ▸) of tin (1.38 Å) and iodine (1.38 Å). Moreover, a closer look at the data in the CIF file reveals a large number of restrictions (58!) and a significant difference between the maximum and minimum residual electron density peaks. These inconsistencies are also noted by *checkCIF* (Spek, 2020 ▸) besides some other alerts that are more formal in nature as some elements are listed but not present in the refinement.

In order to verify the crystal structure with regard to the actual tin–iodide distance, tri­benzyl­tin(IV) iodide was synthesized using a different method from that described in the literature. Single crystals suitable for SCXRD were obtained by recrystallization from ethanol.

**Figure 1:**
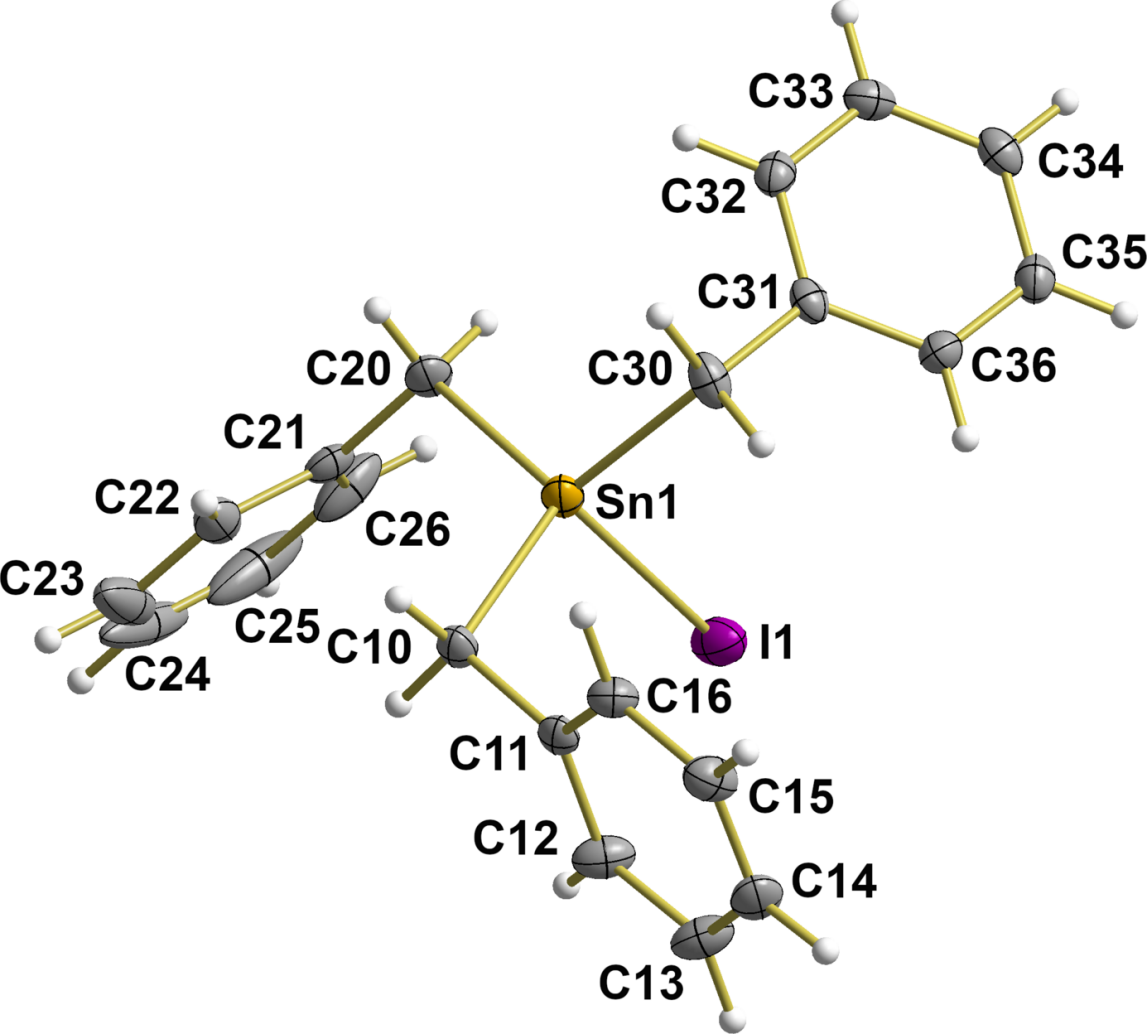
The mol­ecular structure of tri­benzyl­tin(IV) iodide, [Sn(C_7_H_7_)_3_I], with atom numbering. With the exception of the hydrogen atoms, which are shown as spheres of arbitrary radius, all other atoms are drawn as anisotropic displacement ellipsoids at the 60% probability level.

**Figure 2:**
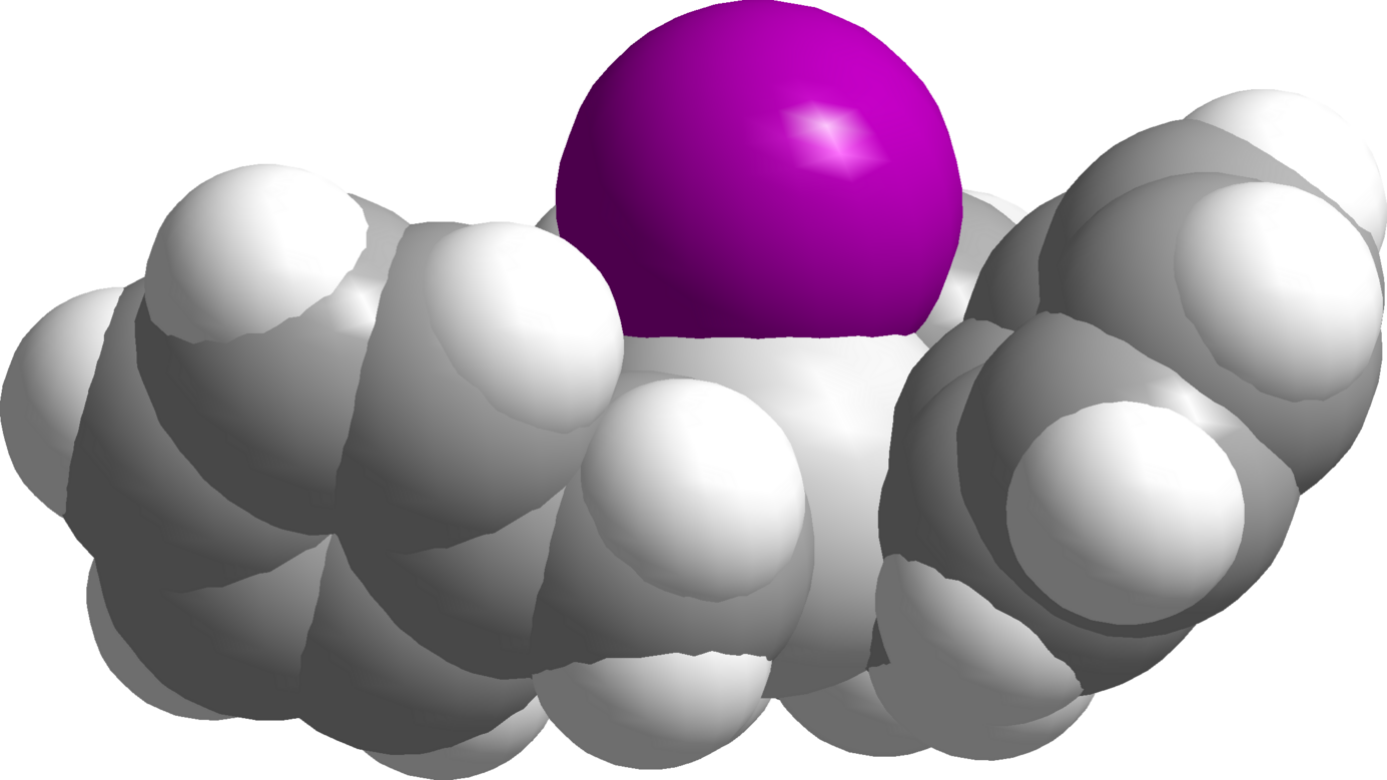
Space-filling model of the tri­benzyl­tin(IV) iodide mol­ecule visualizing its umbrella-like shape; colour code and van der Waals radii used: Sn = bronze, 2.17 Å; I = violet, 1.98 Å; C = dark grey, 1.70; H = white, 1.20 Å.

At T = 100 (2) K, the title compound crystallizes in the polar monoclinic space group *Cc* with four mol­ecules in the unit cell. With a Flack parameter of −0.001 (5), a twin refinement was not necessary. The asymmetric unit comprises one mol­ecule with all atoms in general positions (Fig. 1 ▸). The mol­ecule adopts the expected umbrella-like structure with the tin–iodide bond as shaft and the phenyl groups as stretched cover (Fig. 2 ▸).

The tin atom is distorted tetra­hedrally coordinated from the iodide atom and the three benzyl groups. The tin-iodide distance of 2.7165 (2) Å is now in accord with the sum of the covalent radii of both atoms and the Sn—I distances observed in other triorganotin(IV) iodides (see above).

The tin–carbon bond lengths are almost identical (Table 1 ▸) and correspond to those observed in the low-temperature structure of tri­benzyl­tin(IV) chloride (Ng, 1997 ▸). The bond angles [113.87 (9) to 115.35 (10)°] between the organic groups are larger than those between the iodide atom and the organic moieties [104.02 (7) to 105.78 (7)°]. In the benzyl moieties, some carbon atoms of a phenyl group exhibit distorted anisotropic displacement parameters. However, all attempts to capture this with a disorder model failed. The atom distances between the *sp*^3^-hybridized carbon atoms of the methyl­ene groups and the *sp*^2^-hybridized carbon atom of the phenyl group are almost identical with a mean value of 1.495 (1) Å but the bond angles show a wider [110.1 (2)–114.0 (2)°] range. Within the almost planar phenyl groups, the carbon–carbon distances vary from 1.380 (9) to 1.398 (4) Å with a mean value of 1.390 (6) Å. Bond angles range from 118.3 (2) to 121.1 (2)° with the smallest one being at the *ipso*-carbon atoms.

**Figure 3:**
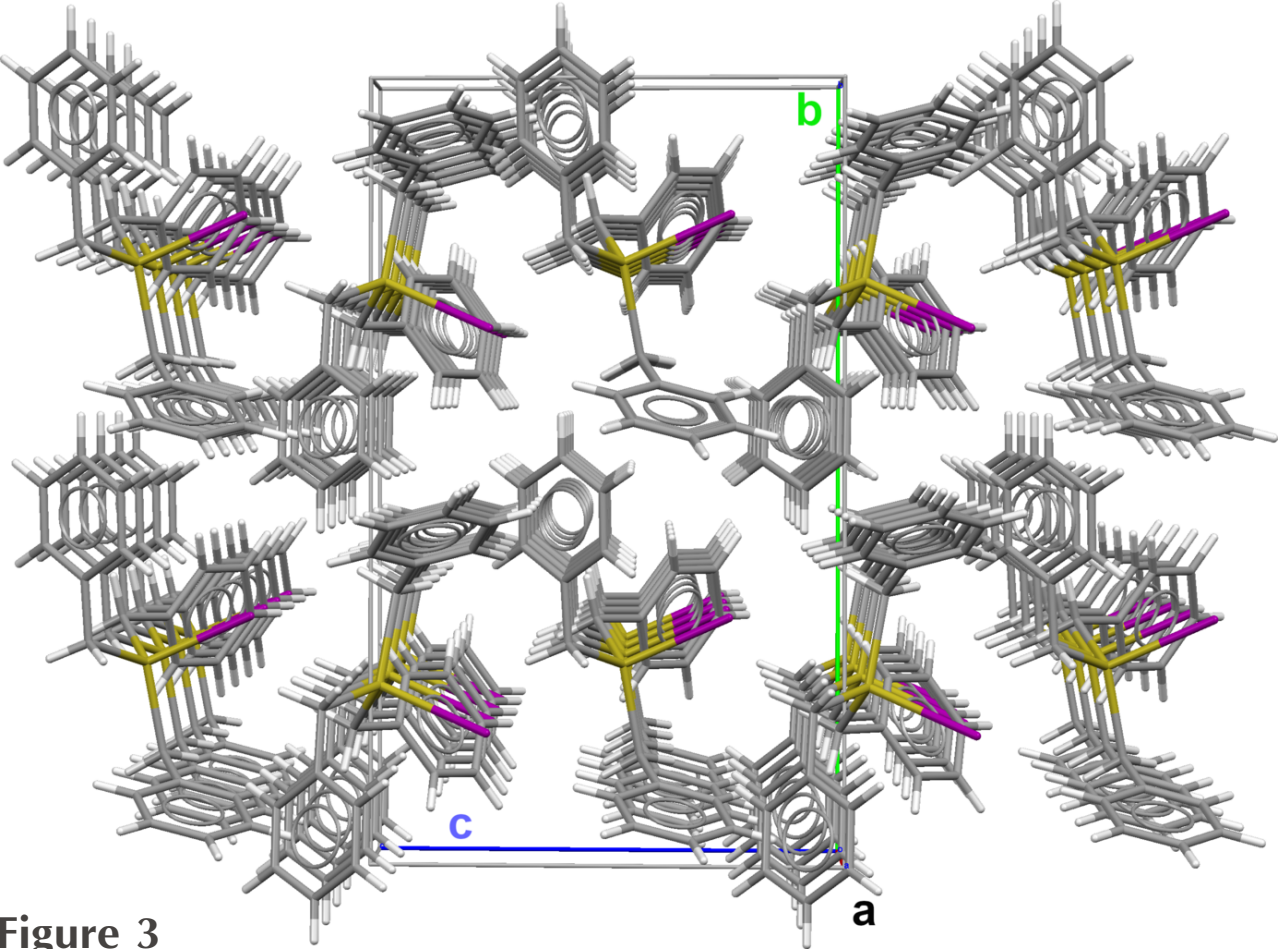
Ball-and-stick model of the tri­benzyl­tin(IV) iodide mol­ecules showing their mol­ecular packing.

**Figure 4:**
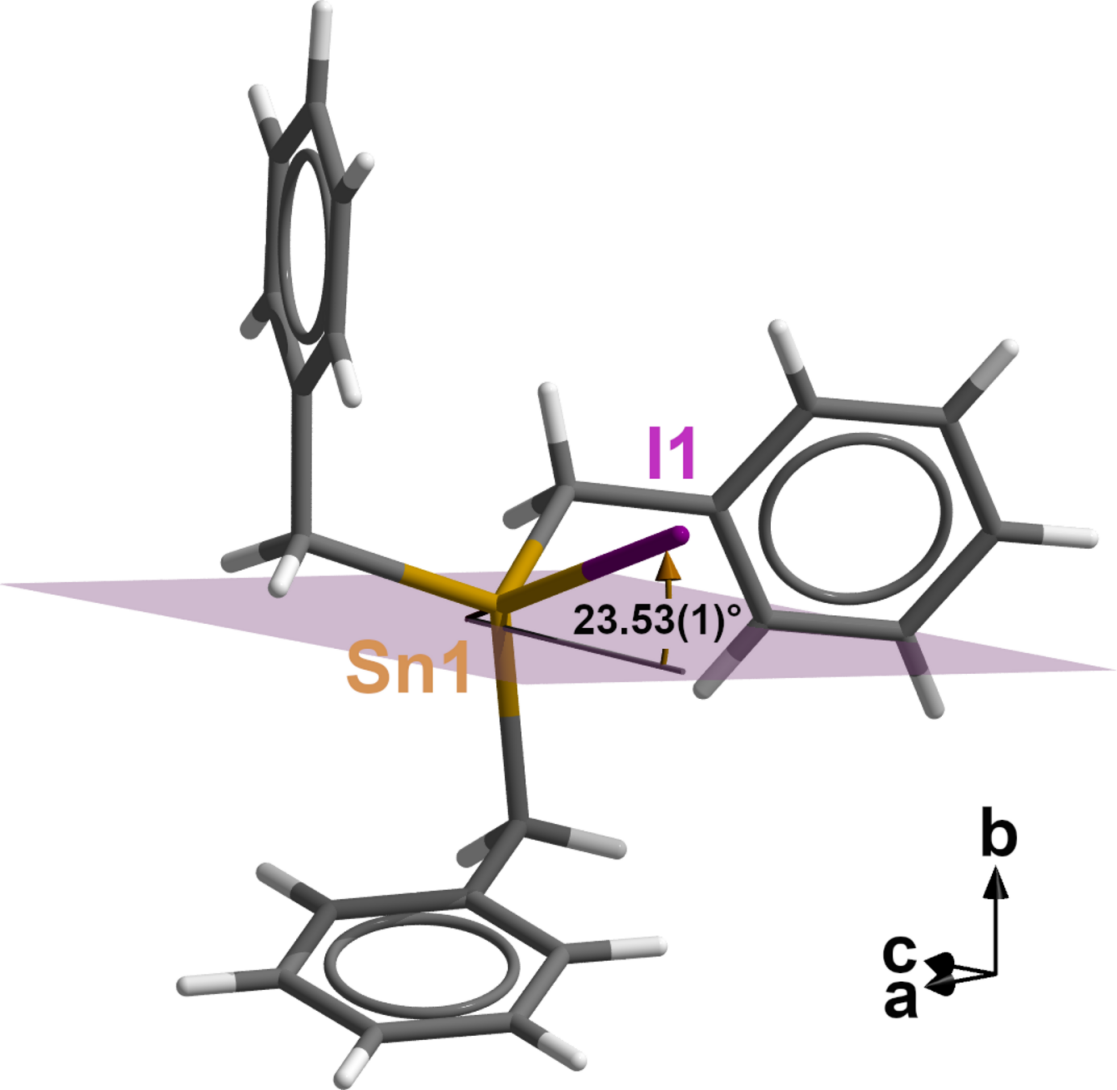
Ball-and-stick model of one tri­benzyl­tin(IV) iodide mol­ecule showing the orientation of the tin–iodide bond with respect to the crystallographic glide plane (pale violet) in direction of the monoclinic *c* axis.

Another inter­esting aspect of the corrected crystal structure of tri­benzyl­tin(IV) iodide concerns the mol­ecular packing (Fig. 3 ▸). Although the dipole moments of the individual mol­ecules are all aligned in the direction of the crystallographic *c* axis, they are not exactly linear as in tri­benzyl­tin(IV) chloride and in the supposed iodide, but at an angle of 23.53 (1)° with respect to the glide plane (Fig. 4 ▸). This means that, unlike in the aforementioned two structures, the halogen atom cannot inter­act with the tin atom of a neighbouring mol­ecule. Thus, the shortest inter­molecular tin–iodide distance is 5.6582 (3) Å. The inter­actions between the mol­ecules are therefore limited solely to dipole–dipole inter­actions between different mol­ecules and van der Waals inter­actions between the atoms of their organic moieties.

The starting point for this study was the unusually short tin–iodide distance reported for tri­benzyl­tin(IV) iodide (Wang *et al.*, 2011 ▸). In fact, this distance corresponds more closely to a tin–bromide distance, which is calculated to be 2.58 Å if a covalent radius of 1.20 Å (Cordero *et al.*, 2008 ▸) is assumed for bromine. At ambient temperature, similar values [2.501 (4)/2.495 (2) Å] are found in Cy_3_SnBr (Howie *et al.*, 2004 ▸) and Ph_3_SnBr (Preut & Huber, 1979 ▸). If it is indeed the crystal structure of the *bromide*, then its crystal structure would be isostructural to that of the room-temperature measurement of tribenzyl tin chloride (Ng, 1997 ▸). However, since it is known that its *c* axis must actually be doubled to obtain the correct structure (Ng, 2009 ▸), this issue should be taken into account when re-investigating the *bromide* (preferably under low-temperature conditions).

## Synthesis and crystallization

While stirring, to a solution of 4.00 g (5 mmol) of hexa­benzyl­distannoxane in ethanol (100 ml), 10 mmol of a 1*M* hydro­iodic acid was added slowly. After stirring for 4 h, the solution was concentrated in a rotary evaporator. The resulting product was recrystallized from ethanol (20 ml). Yield 3.51 g (= 61.6%).

## Refinement

Crystal data, data collection and structure refinement details are summarized in Table 2 ▸.

## Supplementary Material

Crystal structure: contains datablock(s) I. DOI: 10.1107/S2414314626002142/tk4121sup1.cif

Structure factors: contains datablock(s) I. DOI: 10.1107/S2414314626002142/tk4121Isup2.hkl

CCDC reference: 2533567

Additional supporting information:  crystallographic information; 3D view; checkCIF report

## Figures and Tables

**Table 1:** Selected geometric parameters (Å, °)

Sn1—C10	2.161 (2)	Sn1—C30	2.165 (3)
Sn1—C20	2.163 (2)	Sn1—I1	2.7165 (2)
			
C10—Sn1—C20	113.87 (9)	C10—Sn1—I1	105.78 (7)
C10—Sn1—C30	111.93 (10)	C20—Sn1—I1	104.59 (7)
C20—Sn1—C30	115.35 (10)	C30—Sn1—I1	104.02 (7)

**Table 2:** Experimental details

Crystal data	
Chemical formula	[Sn(C_7_H_7_)_3_I]
*M*_r_	518.97
Crystal system, space group	Monoclinic, *Cc*
Temperature (K)	100
*a*, *b*, *c* (Å)	9.5368 (3), 18.3884 (7), 11.1992 (4)
β (°)	98.475 (2)
*V* (Å^3^)	1942.52 (12)
*Z*	4
Radiation type	Mo *K*α
μ (mm^−1^)	2.90
Crystal size (mm)	0.36 × 0.28 × 0.10

Data collection	
Diffractometer	Bruker APEXII CCD area detector
Absorption correction	Multi-scan (*SADABS*; Krause *et al.*, 2015 ▸)
*T*_min_, *T*_max_	0.457, 0.701
No. of measured, independent and observed [*I* > 2σ(*I*)] reflections	65839, 4688, 4675
*R*_int_	0.028
(sin θ/λ)_max_ (Å^−1^)	0.660

Refinement	
*R*[*F*^2^ > 2σ(*F*^2^)], *wR*(*F*^2^), *S*	0.011, 0.028, 1.06
No. of reflections	4688
No. of parameters	214
No. of restraints	2
H-atom treatment	Only H-atom displacement parameters refined
Δρ_max_, Δρ_min_ (e Å^−3^)	0.44, −0.38
Absolute structure	Flack *x* determined using 2320 quotients [(*I*^+^)−(*I*^−^)]/[(*I*^+^)+(*I*^−^)] (Parsons *et al.*, 2013 ▸)
Absolute structure parameter	−0.001 (5)

Computer programs: *APEX2* and *SAINT* (Bruker, 2009 ▸), *SHELXS97* (Sheldrick, 2008 ▸), *SHELXL2014/7* (Sheldrick, 2015 ▸), *DIAMOND* (Brandenburg, 2006 ▸), *Mercury* (Macrae *et al.*, 2020 ▸) and *publCIF* (Westrip, 2010 ▸).

## full crystallographic data

### Tribenzyliodidotin(IV) Crystal data

**Table:**

[Sn(C_7_H_7_)_3_I]	*F*(000) = 1000
*M**_r_* = 518.97	*D*_x_ = 1.775 Mg m^−^^3^
Monoclinic, *C**c*	Mo *K*α radiation, λ = 0.71073 Å
*a* = 9.5368 (3) Å	Cell parameters from 9937 reflections
*b* = 18.3884 (7) Å	θ = 2.4–31.5°
*c* = 11.1992 (4) Å	µ = 2.90 mm^−^^1^
β = 98.475 (2)°	*T* = 100 K
*V* = 1942.52 (12) Å^3^	Plate, colourless
*Z* = 4	0.36 × 0.28 × 0.10 mm

### Tribenzyliodidotin(IV) Data collection

**Table:**

Bruker APEXII CCD area detector diffractometer	4675 reflections with *I* > 2σ(*I*)
phi and ω scans	*R*_int_ = 0.028
Absorption correction: multi-scan (SADABS; Krause *et al.*, 2015)	θ_max_ = 28.0°, θ_min_ = 2.2°
*T*_min_ = 0.457, *T*_max_ = 0.701	*h* = −12→12
65839 measured reflections	*k* = −24→24
4688 independent reflections	*l* = −14→14

### Tribenzyliodidotin(IV) Refinement

**Table:**

Refinement on *F*^2^	Hydrogen site location: inferred from neighbouring sites
Least-squares matrix: full	Only H-atom displacement parameters refined
*R*[*F*^2^ > 2σ(*F*^2^)] = 0.011	*w* = 1/[σ^2^(*F*_o_^2^) + (0.0162*P*)^2^ + 0.9433*P*] where *P* = (*F*_o_^2^ + 2*F*_c_^2^)/3
*wR*(*F*^2^) = 0.028	(Δ/σ)_max_ = 0.001
*S* = 1.06	Δρ_max_ = 0.44 e Å^−^^3^
4688 reflections	Δρ_min_ = −0.38 e Å^−^^3^
214 parameters	Absolute structure: Flack *x* determined using 2320 quotients [(*I*^+^)-(*I*^-^)]/[(*I*^+^)+(*I*^-^)] (Parsons *et al.*, 2013)
2 restraints	Absolute structure parameter: −0.001 (5)
Primary atom site location: structure-invariant direct methods	

### Tribenzyliodidotin(IV) Special details

**Table:**

Geometry. All esds (except the esd in the dihedral angle between two l.s. planes) are estimated using the full covariance matrix. The cell esds are taken into account individually in the estimation of esds in distances, angles and torsion angles; correlations between esds in cell parameters are only used when they are defined by crystal symmetry. An approximate (isotropic) treatment of cell esds is used for estimating esds involving l.s. planes.
Refinement. The H atoms were placed geometrically and allowed to ride on the C-atom with d(C—H) = 0.95–0.99 Å) and common *U*_iso_ = 1.2*U*_eq_(C) parameters.

### Tribenzyliodidotin(IV) Fractional atomic coordinates and isotropic or equivalent isotropic displacement parameters (Å^2^)

**Table:**

	*x*	*y*	*z*	*U*_iso_*/*U*_eq_	
Sn1	0.35288 (2)	0.26006 (2)	0.46465 (2)	0.01568 (4)	
I1	0.37306 (2)	0.31903 (2)	0.24543 (2)	0.02400 (4)	
C10	0.5495 (3)	0.28642 (13)	0.5783 (2)	0.0191 (5)	
H10A	0.5486	0.2662	0.6601	0.041 (7)*	
H10B	0.6298	0.2643	0.5446	0.041 (7)*	
C11	0.5686 (2)	0.36712 (13)	0.5861 (2)	0.0171 (4)	
C12	0.6418 (3)	0.40335 (15)	0.5048 (2)	0.0251 (5)	
H12	0.6833	0.3762	0.4469	0.031 (4)*	
C13	0.6549 (4)	0.47872 (16)	0.5072 (3)	0.0284 (6)	
H13	0.7045	0.5027	0.4510	0.031 (4)*	
C14	0.5957 (3)	0.51872 (14)	0.5916 (3)	0.0260 (5)	
H14	0.6027	0.5703	0.5923	0.031 (4)*	
C15	0.5262 (3)	0.48346 (15)	0.6751 (3)	0.0259 (5)	
H15	0.4883	0.5108	0.7348	0.031 (4)*	
C16	0.5115 (3)	0.40829 (14)	0.6719 (2)	0.0212 (5)	
H16	0.4622	0.3847	0.7288	0.031 (4)*	
C20	0.3233 (3)	0.14495 (13)	0.4297 (2)	0.0222 (5)	
H20A	0.3012	0.1206	0.5036	0.026 (6)*	
H20B	0.2416	0.1378	0.3653	0.026 (6)*	
C21	0.4520 (3)	0.11064 (13)	0.3922 (2)	0.0206 (5)	
C22	0.5703 (3)	0.09653 (15)	0.4778 (3)	0.0303 (6)	
H22	0.5689	0.1090	0.5599	0.065 (6)*	
C23	0.6900 (4)	0.06432 (18)	0.4439 (5)	0.0578 (13)	
H23	0.7707	0.0549	0.5024	0.065 (6)*	
C24	0.6909 (6)	0.04590 (18)	0.3234 (7)	0.0754 (19)	
H24	0.7726	0.0241	0.2994	0.065 (6)*	
C25	0.5732 (7)	0.05930 (19)	0.2389 (5)	0.0687 (17)	
H25	0.5741	0.0461	0.1570	0.065 (6)*	
C26	0.4534 (4)	0.09175 (15)	0.2721 (3)	0.0399 (8)	
H26	0.3730	0.1010	0.2133	0.065 (6)*	
C30	0.1740 (3)	0.31566 (14)	0.5217 (2)	0.0229 (5)	
H30A	0.1526	0.2928	0.5970	0.027 (5)*	
H30B	0.2008	0.3669	0.5404	0.027 (5)*	
C31	0.0428 (3)	0.31461 (13)	0.4302 (2)	0.0176 (5)	
C32	−0.0478 (3)	0.25443 (13)	0.4165 (3)	0.0198 (5)	
H32	−0.0246	0.2125	0.4651	0.035 (5)*	
C33	−0.1707 (3)	0.25487 (14)	0.3334 (2)	0.0216 (5)	
H33	−0.2314	0.2136	0.3256	0.035 (5)*	
C34	−0.2051 (3)	0.31582 (14)	0.2614 (3)	0.0216 (5)	
H34	−0.2893	0.3164	0.2043	0.027 (5)*	
C35	−0.1153 (3)	0.37591 (14)	0.2734 (2)	0.0216 (5)	
H35	−0.1383	0.4176	0.2243	0.035 (5)*	
C36	0.0068 (3)	0.37513 (14)	0.3565 (2)	0.0203 (5)	
H36	0.0675	0.4164	0.3637	0.035 (5)*	

### Tribenzyliodidotin(IV) Atomic displacement parameters (Å^2^)

**Table:**

	*U*^11^	*U*^22^	*U*^33^	*U*^12^	*U*^13^	*U*^23^
Sn1	0.01384 (7)	0.01310 (6)	0.01906 (7)	−0.00108 (6)	−0.00099 (5)	−0.00084 (6)
I1	0.02107 (7)	0.02905 (8)	0.02086 (7)	−0.00482 (6)	−0.00026 (5)	0.00397 (6)
C10	0.0155 (11)	0.0181 (11)	0.0222 (11)	−0.0007 (9)	−0.0026 (9)	−0.0010 (9)
C11	0.0138 (10)	0.0175 (11)	0.0185 (11)	−0.0019 (8)	−0.0030 (8)	−0.0014 (8)
C12	0.0289 (13)	0.0258 (13)	0.0223 (13)	−0.0093 (11)	0.0092 (10)	−0.0080 (10)
C13	0.0337 (14)	0.0267 (13)	0.0255 (13)	−0.0129 (12)	0.0071 (11)	−0.0011 (11)
C14	0.0252 (13)	0.0174 (12)	0.0341 (14)	−0.0067 (10)	−0.0003 (11)	−0.0032 (10)
C15	0.0227 (12)	0.0220 (13)	0.0337 (14)	−0.0027 (10)	0.0068 (11)	−0.0090 (11)
C16	0.0192 (11)	0.0227 (12)	0.0223 (11)	−0.0040 (10)	0.0046 (9)	−0.0013 (10)
C20	0.0201 (12)	0.0148 (11)	0.0304 (13)	−0.0046 (9)	−0.0006 (10)	−0.0037 (9)
C21	0.0263 (12)	0.0121 (10)	0.0245 (12)	−0.0058 (9)	0.0072 (10)	−0.0007 (9)
C22	0.0230 (15)	0.0177 (13)	0.0492 (18)	−0.0021 (10)	0.0016 (13)	0.0002 (12)
C23	0.0243 (16)	0.0217 (15)	0.130 (4)	−0.0004 (13)	0.019 (2)	0.001 (2)
C24	0.068 (3)	0.0166 (15)	0.163 (6)	−0.0026 (18)	0.088 (4)	−0.004 (2)
C25	0.126 (5)	0.0201 (16)	0.081 (3)	−0.008 (2)	0.083 (3)	−0.0058 (18)
C26	0.077 (3)	0.0176 (13)	0.0295 (15)	−0.0047 (14)	0.0210 (16)	−0.0007 (11)
C30	0.0199 (13)	0.0260 (13)	0.0213 (13)	0.0068 (10)	−0.0020 (10)	−0.0041 (10)
C31	0.0157 (11)	0.0218 (12)	0.0153 (11)	0.0042 (9)	0.0028 (9)	−0.0005 (8)
C32	0.0218 (13)	0.0188 (11)	0.0200 (12)	0.0033 (9)	0.0067 (10)	0.0027 (9)
C33	0.0185 (12)	0.0219 (12)	0.0254 (13)	−0.0037 (9)	0.0063 (10)	−0.0051 (9)
C34	0.0179 (12)	0.0269 (13)	0.0200 (12)	0.0036 (9)	0.0024 (10)	−0.0032 (9)
C35	0.0226 (12)	0.0203 (11)	0.0219 (13)	0.0046 (10)	0.0033 (10)	0.0032 (9)
C36	0.0205 (12)	0.0178 (11)	0.0230 (12)	−0.0016 (9)	0.0041 (9)	0.0014 (9)

### Tribenzyliodidotin(IV) Geometric parameters (Å, º)

**Table:**

Sn1—C10	2.161 (2)	C22—C23	1.388 (5)
Sn1—C20	2.163 (2)	C22—H22	0.9500
Sn1—C30	2.165 (3)	C23—C24	1.393 (8)
Sn1—I1	2.7165 (2)	C23—H23	0.9500
C10—C11	1.496 (3)	C24—C25	1.380 (9)
C10—H10A	0.9900	C24—H24	0.9500
C10—H10B	0.9900	C25—C26	1.387 (6)
C11—C16	1.396 (3)	C25—H25	0.9500
C11—C12	1.396 (3)	C26—H26	0.9500
C12—C13	1.391 (4)	C30—C31	1.496 (3)
C12—H12	0.9500	C30—H30A	0.9900
C13—C14	1.382 (4)	C30—H30B	0.9900
C13—H13	0.9500	C31—C36	1.398 (3)
C14—C15	1.384 (4)	C31—C32	1.398 (4)
C14—H14	0.9500	C32—C33	1.385 (4)
C15—C16	1.389 (4)	C32—H32	0.9500
C15—H15	0.9500	C33—C34	1.391 (4)
C16—H16	0.9500	C33—H33	0.9500
C20—C21	1.494 (4)	C34—C35	1.392 (4)
C20—H20A	0.9900	C34—H34	0.9500
C20—H20B	0.9900	C35—C36	1.380 (4)
C21—C26	1.391 (4)	C35—H35	0.9500
C21—C22	1.393 (4)	C36—H36	0.9500
			
C10—Sn1—C20	113.87 (9)	C23—C22—C21	120.5 (4)
C10—Sn1—C30	111.93 (10)	C23—C22—H22	119.8
C20—Sn1—C30	115.35 (10)	C21—C22—H22	119.8
C10—Sn1—I1	105.78 (7)	C22—C23—C24	119.4 (4)
C20—Sn1—I1	104.59 (7)	C22—C23—H23	120.3
C30—Sn1—I1	104.02 (7)	C24—C23—H23	120.3
C11—C10—Sn1	110.14 (15)	C25—C24—C23	120.0 (3)
C11—C10—H10A	109.6	C25—C24—H24	120.0
Sn1—C10—H10A	109.6	C23—C24—H24	120.0
C11—C10—H10B	109.6	C24—C25—C26	120.8 (4)
Sn1—C10—H10B	109.6	C24—C25—H25	119.6
H10A—C10—H10B	108.1	C26—C25—H25	119.6
C16—C11—C12	118.3 (2)	C25—C26—C21	119.5 (4)
C16—C11—C10	121.5 (2)	C25—C26—H26	120.3
C12—C11—C10	120.2 (2)	C21—C26—H26	120.3
C13—C12—C11	121.0 (2)	C31—C30—Sn1	114.04 (17)
C13—C12—H12	119.5	C31—C30—H30A	108.7
C11—C12—H12	119.5	Sn1—C30—H30A	108.7
C14—C13—C12	119.9 (2)	C31—C30—H30B	108.7
C14—C13—H13	120.0	Sn1—C30—H30B	108.7
C12—C13—H13	120.0	H30A—C30—H30B	107.6
C13—C14—C15	119.8 (2)	C36—C31—C32	118.1 (2)
C13—C14—H14	120.1	C36—C31—C30	120.1 (2)
C15—C14—H14	120.1	C32—C31—C30	121.7 (2)
C14—C15—C16	120.4 (2)	C33—C32—C31	121.1 (2)
C14—C15—H15	119.8	C33—C32—H32	119.4
C16—C15—H15	119.8	C31—C32—H32	119.4
C15—C16—C11	120.6 (2)	C32—C33—C34	119.9 (2)
C15—C16—H16	119.7	C32—C33—H33	120.1
C11—C16—H16	119.7	C34—C33—H33	120.1
C21—C20—Sn1	111.94 (16)	C33—C34—C35	119.6 (3)
C21—C20—H20A	109.2	C33—C34—H34	120.2
Sn1—C20—H20A	109.2	C35—C34—H34	120.2
C21—C20—H20B	109.2	C36—C35—C34	120.2 (2)
Sn1—C20—H20B	109.2	C36—C35—H35	119.9
H20A—C20—H20B	107.9	C34—C35—H35	119.9
C26—C21—C22	119.8 (3)	C35—C36—C31	121.0 (2)
C26—C21—C20	120.2 (3)	C35—C36—H36	119.5
C22—C21—C20	120.0 (2)	C31—C36—H36	119.5
			
Sn1—C10—C11—C16	86.8 (2)	C22—C23—C24—C25	−0.4 (5)
Sn1—C10—C11—C12	−91.7 (2)	C23—C24—C25—C26	0.6 (5)
C16—C11—C12—C13	−1.5 (4)	C24—C25—C26—C21	−0.3 (5)
C10—C11—C12—C13	177.0 (3)	C22—C21—C26—C25	−0.3 (4)
C11—C12—C13—C14	0.4 (5)	C20—C21—C26—C25	−179.4 (3)
C12—C13—C14—C15	1.4 (5)	Sn1—C30—C31—C36	−99.7 (2)
C13—C14—C15—C16	−2.1 (4)	Sn1—C30—C31—C32	81.2 (3)
C14—C15—C16—C11	1.1 (4)	C36—C31—C32—C33	−0.8 (4)
C12—C11—C16—C15	0.7 (4)	C30—C31—C32—C33	178.4 (2)
C10—C11—C16—C15	−177.8 (2)	C31—C32—C33—C34	0.4 (4)
Sn1—C20—C21—C26	−105.3 (2)	C32—C33—C34—C35	0.1 (4)
Sn1—C20—C21—C22	75.6 (3)	C33—C34—C35—C36	−0.2 (4)
C26—C21—C22—C23	0.6 (4)	C34—C35—C36—C31	−0.2 (4)
C20—C21—C22—C23	179.7 (2)	C32—C31—C36—C35	0.7 (4)
C21—C22—C23—C24	−0.2 (4)	C30—C31—C36—C35	−178.5 (2)
